# Neonatal Abstinence Syndrome: An Insight Over Impact of Maternal Substance Use

**DOI:** 10.7759/cureus.47980

**Published:** 2023-10-30

**Authors:** Omkar Dumbhare, Amar Taksande

**Affiliations:** 1 Pediatrics, Jawaharlal Nehru Medical College, Datta Meghe Institute of Higher Education and Research, Wardha, IND

**Keywords:** developmental impacts, multidisciplinary care, withdrawal symptoms, opioid crisis, maternal substance use, neonatal abstinence syndrome

## Abstract

Neonatal abstinence syndrome (NAS) highlights the intricate interplay between maternal substance use during pregnancy and the challenges neonates face from the distressing global opioid crisis. This comprehensive review captures the multilayered landscape of NAS, encircling its underlying mechanisms, epidemiology, diagnostic intricacies, clinical manifestations, continuing developmental impacts, treatment paradigms, and the crucial role of multidisciplinary care. The core pathophysiology of NAS involves the transplacental passage of addictive substances, activating chemical dependence in the maturing fetus, which is characterized by neurotransmitter dysregulation, neuroadaptations, and receptor sensitization. A diverse clinical presentation ranges from central nervous system hyperactivity and autonomic dysregulation to gastrointestinal manifestations, necessitating homogenous assessment tools such as the Finnegan Neonatal Abstinence Scoring System. The demand for a multilayered approach is essential for comprehensive management, involving pharmacological interventions like morphine or methadone and non-pharmacological strategies such as swaddling. The complications of NAS are not only limited to but are also well beyond infancy, leading to behavioral, longstanding cognitive, and socioemotional consequences. Addressing these developmental arcs demands decisive longitudinal monitoring and early interventions. NAS management is fundamentally multidisciplinary, requiring the teamwork of nurses, social workers, psychologists, pediatricians, and neonatologists. Apart from the clinical realm, managing the psychosocial needs of families traversing NAS requires resources and empathy. A crucial comprehensive approach is essential to confront the challenges and limitations of NAS. From early identification and prevention to longstanding support through pharmacological, non-pharmacological, and psychological channels, it creates a holistic structure that emerges as the basis for understanding the complicated relationship between maternal substance use and its impact on neonates. An amalgamation of community engagement, society, policy initiatives, and medical expertise is essential to mitigate the repercussions of NAS and adopt healthier outcomes for affected infants.

## Introduction and background

Neonatal abstinence syndrome (NAS), withdrawal symptoms experienced by newborns due to exposure to addictive substances during gestation, is a multilayered and complex condition primarily linked to maternal substance abuse. An intricate relationship between the maternal environment and the vulnerable developing fetus is characterized by NAS manifesting neurological and physical symptoms. The ever-increasing occurrence of NAS emphasizes its rise as a persistent public health concern, affecting maternal and neonatal health [1]. Healthcare systems deal with the multidimensional challenges modeled by NAS, entailing a thorough understanding and intervention strategies. Apart from medical realms, NAS compasses across ethical, social, and economic dimensions, urging attention from researchers, clinicians, society, and policymakers. The global opioid crisis comprises factors ranging from illicit drug use to prescription practices, developing an alarming rise in opioid addiction [2]. This crisis has unconsciously promoted NAS incidence, as maternal substance use during pregnancy disturbs the fetal environment, resulting in complex clinical manifestations of NAS. These include disturbances in equilibrium for optimal growth and neurological development, low birth weight, preterm births, and placental complications, further highlighting the requirement for comprehensive measures [3].

## Review

Methods

This systematic review strictly followed the guidelines outlined in the Preferred Reporting Items for Systematic Reviews and Meta-Analyses (PRISMA). Data collection occurred in August 2023 by searching various electronic databases, including PubMed, Google Scholar, ScienceDirect, PubMed Central, and Cochrane Data. These searches involved the utilization of keywords like "Neonatal Abstinence Syndrome," "maternal substance abuse," "opioid crisis," "withdrawal symptoms," and "multidisciplinary care," both independently and in various combinations. A total of 2191 cases were identified through these keyword-based searches. Subsequently, the articles were assessed based on their titles, abstracts, and relevance to the research inquiry. To further refine the selection, inclusion and exclusion criteria were applied to identify articles pertinent to the study. The databases were most recently accessed in August 2023.

Search Sources/Search Strategy

In PubMed, Google Scholar, ScienceDirect, PubMed Central, and Cochrane data, we used the Medical Subject Headings (MeSH) strategy we obtained: ("Neonatal Abstinence syndrome/maternal substance use" [Majr] OR "Neonatal Abstinence syndrome/clinical presentation" [Majr] OR "Neonatal Abstinence syndrome/genetics" [Majr] OR "Neonatal Abstinence syndrome/diagnosis" [Majr] OR "Neonatal Abstinence syndrome/developmental outcomes" [Majr] OR "Neonatal Abstinence syndrome/long term effects" [Majr]); for the treatment, it was ("Neonatal Abstinence syndrome treatment/non-pharmacological" [Majr] OR "Abstinence syndrome treatment/pharmacological" [Majr] OR "Abstinence syndrome treatment/physical therapy" [Majr] OR "Abstinence syndrome treatment/occupational therapy" [Majr] OR "Abstinence syndrome treatment/multidisciplinary care" [Majr] OR "Abstinence syndrome treatment/support" [Majr] OR "Abstinence syndrome treatment/ethical considerations" [Majr]). We obtained the most pertinent research papers and used them in different arrangements with the Booleans "AND," "OR."

Inclusion and Exclusion Criteria

Our selection criteria primarily encompassed English-language papers published in the past decade that directly pertained to the core inquiries of this review article. We specifically sought systematic reviews, randomized clinical trials, and observational studies. Conversely, papers written in languages other than English, those unrelated to the research questions, and those addressing subjects beyond the scope of NAS are excluded from consideration.

Results

**Figure 1:**
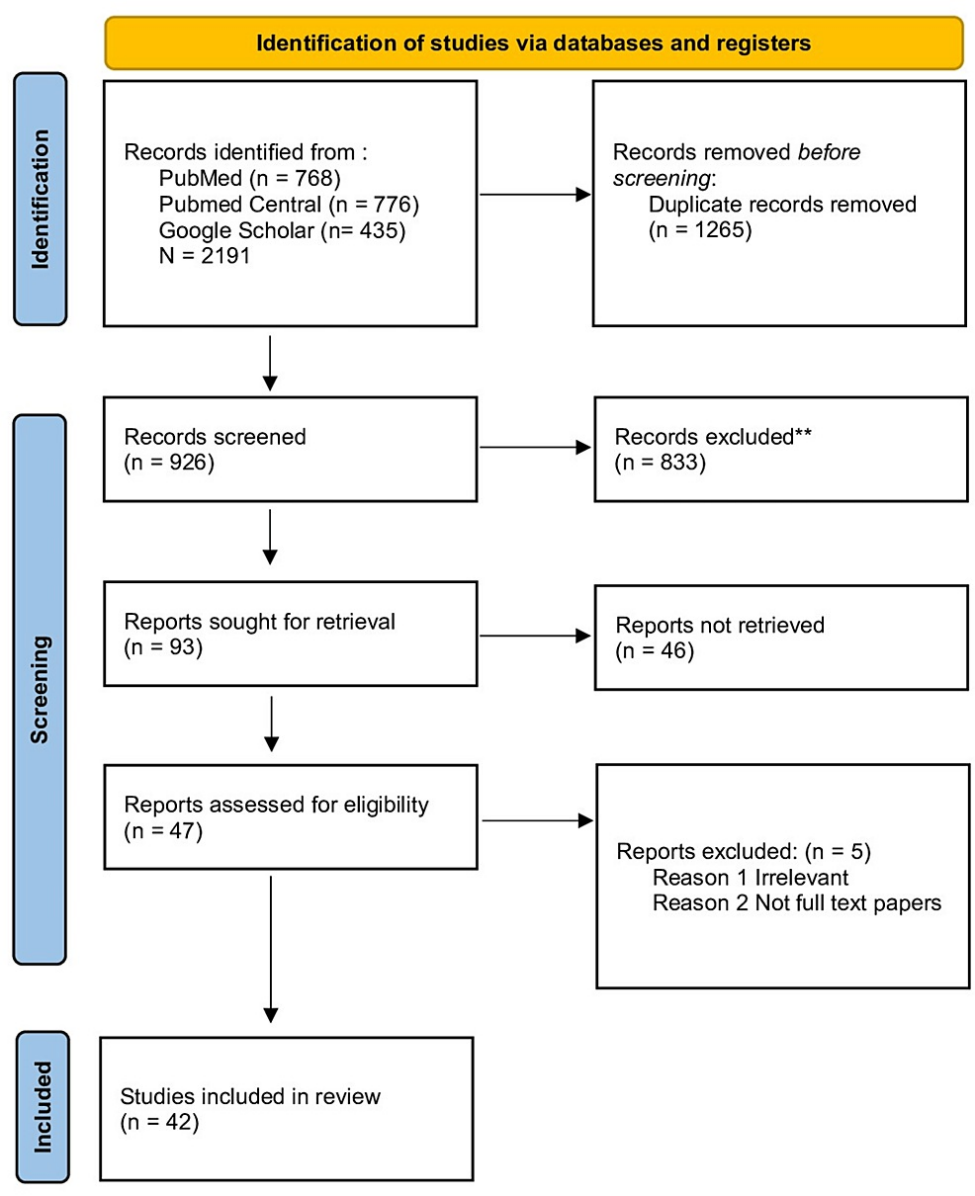
PRISMA 2020 flow diagram for the systematic review. PRISMA: Preferred Reporting Items for Systematic Reviews and Meta-Analyses; RCT: randomized controlled trial. **Of 833 records, 67 were excluded by the author and 766 were excluded by using an automation tool (Cochrane RCT Classifier).

A total of 2191 studies were initially identified from databases. However, after applying filters based on inclusion criteria, such as being in English, articles from the last 24 years, and involving humans, clinical trials, all types of reviews, and observational studies, the number of studies was reduced by 926. After further screening and quality appraisal, the number of studies included was reduced to 42. The study found that 30 studies provided evidence of a relationship between NAS, opioid crisis, withdrawal symptoms, and developmental impacts, with 14 studies specifically finding that maternal substance abuse manifests neurological and physiological symptoms in developing fetuses. Figure 1 summarizes the overall methodology, mentioning screening, included, and excluded data involved in this study.

Pathophysiology of NAS

**Figure 2:**
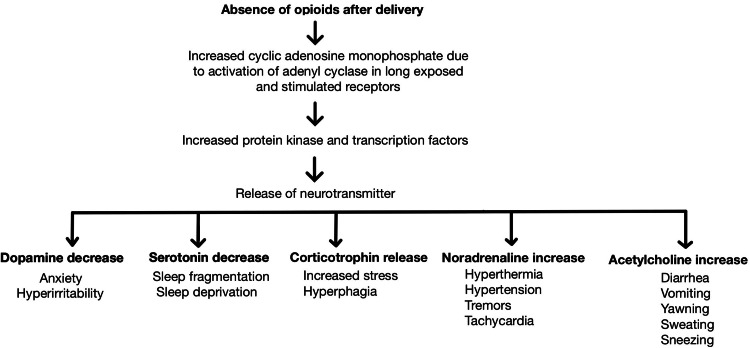
Pathophysiology of NAS along with various clinical features. NAS: neonatal abstinence syndrome. Source: Authors.

The pathophysiology of NAS involves the transplacental passage of addictive substances, disrupting the equilibrium essential for fetal development. Opioids cross the placental barrier due to their lipophilic properties and bind to fetal opioid receptors, triggering a cascade of neuroadaptations. The developing fetus responds to such continuous exposure by altering gene expression and receptor density, ultimately leading to functional changes in the fetal brain [4]. Peripheral organs are also affected, other than the central nervous system, due to these neuroadaptations. The autonomic nervous and endocrine systems change, causing variations in feeding behavior and impaired thermoregulation. This complicated relationship between maternal substance abuse and fetal adaptations forms the groundwork of NAS's complex pathophysiology [5]. Another critical mechanism involved is neurotransmitter dysregulation, where prenatal exposure to opioids causes a severe imbalance of neurotransmitters, contributing to withdrawal symptoms. A core neurotransmitter, dopamine, experiences abnormal fluctuations. Perinatal exposure to opioids leads to a surge in dopamine release, and its abrupt cessation results in a dopamine deficit, leading to characteristic tremors, irritability, and desolate crying seen in NAS. Serotonin and norepinephrine also play vital roles. Prenatal opioid exposure leads to imbalances in these neurotransmitters, elevating the clinical manifestation of NAS symptoms [6]. Prolonged exposure to opioids in utero provokes the maturing fetal brain to acclimatize receptor sensitization and downregulation. In contrast, the chronic presence of opioids leads to an increase in receptor sensitivity and density to counteract the depressant effects of these substances. This adaptive mechanism boosts the fetal brain's sensitivity to opioids, preserving the cycle of dependence. When the opioid supply is terminated from the mother upon birth, the receptor's sensitivity is heightened, triggering an augmented response to the absence of opioids. This heightened response explains NAS's hyperexcitability and withdrawal symptoms [7,8]. Figure 2 summarizes the overall pathophysiology of NAS along with various leading clinical features.

Maternal substance use and NAS

Due to the physiochemical properties of substances such as opioids, benzodiazepines, and cocaine, they readily cross the placenta [9]. The placenta, even though a protective barrier, is not impervious to the transference of addictive substances, leading to exposure of these substances to fetal circulation and accumulation within the fetal tissues [10]. Opioids are a small and lipophilic class of potent analgesics. Upon entering the fetal circulation, they interact and bind to mu-opioid receptors in the maturing fetal brain [11]. Within the fetal brain, mu-opioid receptors are coupled to G-proteins, which trigger intracellular signaling cascades upon ligand binding. The presence of opioids activates these receptors, causing the release of neurotransmitters and cellular responses. Prolonged opioid exposure alters receptor density and gene expression, developing a state of physiological dependence where the fetal neural network becomes habituated to continuous opioid stimulation [12]. Precise molecular signaling, such as synaptogenesis, myelination, and neuronal development, is complex. Disruption in these processes leads to abnormalities in neural circuitry. Opioids obstruct the production and migration of neural progenitor cells, altering neural connectivity and function [13]. Abrupt cessation of these substances at birth disrupts the fetal environment, dismantling the chemical equilibrium the neonate has adapted to, resulting in withdrawal symptoms [14].

Clinical presentation and diagnosis

Considering the infant's medical history and the presence of characteristic withdrawal signs, careful clinical evaluation is vital for diagnosing NAS. The range of NAS symptoms includes various neurological and physiological manifestations, providing an understanding of the complex physiological disruptions resulting from the sudden removal of opioids that the fetus has adapted during gestation [15]. Gastrointestinal manifestations are conspicuous features of NAS, including poor weight gain and feeding difficulties, vomiting, nausea, and diarrhea. These symptoms arise from the imbalance and dominance of the autonomic nervous system's control over gastrointestinal motility and function [16]. Autonomic symptoms, the hallmark of NAS, highlight the extreme perturbations within the autonomic nervous system. Sweating, sneezing, fever, yawning, and skin mottling emphasize impaired autonomic responses. The abrupt withdrawal of opioids disturbs the balance between sympathetic and parasympathetic tone, leading to a dysregulated autonomic response seen in these infants [17]. Hyperactivity of the central nervous system is evident through symptoms like high-pitched crying, tremors, irritability, and an exaggerated Moro reflex. Dopamine and serotonin are the chief neurotransmitters which, in the absence of opioids, are dysregulated, resulting in central nervous system clinical manifestations. These symptoms represent the neonate's struggle to adapt to the sudden change in neurotransmitter balance, leading to neuronal hyperexcitability [18,19].

Due to the overlapping symptoms of various other pathologies and delayed onset, the diagnosis of NAS is complex. Clinicians must differentiate NAS from other medical conditions with similar clinical features, such as metabolic disorders or sepsis. Also, a maternal history of substance use may only sometimes be reliable. Therefore, a comprehensive assessment, including appropriate laboratory tests, maternal history, and expert clinical judgment, is vital for an accurate diagnosis [20]. The scoring system, such as the Finnegan and Neonatal Withdrawal Inventory, helps regulate NAS assessment. Specific signs and symptoms are assigned scores, simplifying monitoring of NAS severity and objective evaluation [21]. The application of such scoring systems supports healthcare providers in making informed clinical decisions regarding the need for pharmacological intervention and the appropriate management strategy [16].

Treatment approaches

The management of NAS includes a multilayered approach meant to support the neonate's physiological stability, address potential longstanding consequences, and alleviate withdrawal symptoms. Contrasting treatment strategies are used based on the maternal history of substance abuse, the severity of NAS, and the presence of comorbidities. Strategies include non-pharmacological, pharmacological, and developmental interventions and family-centered care [15]. Non-pharmacological interventions involve swaddling and environmental modification. Provision and maintenance of low noise levels, lighting, and tactile stimulation help reduce the neonate's distress. Assistance in diminishing the overstimulation, as seen in NAS-affected infants, can be done by recreating a sensory environment similar to that of the womb. Optimizing the neonate's nutritional intake is essential and tapers withdrawal symptoms. Breastfeeding is encouraged as breast milk contains maternal opioids along with essential nutrients. The presence of maternal opioids aids in tapering withdrawal symptoms. Mandatory close monitoring is crucial due to probable variability in opioid content [22]. Pharmacological interventions involve using various drugs such as morphine, methadone, and buprenorphine. The most commonly used agents to treat severe cases of NAS are morphine and methadone. Administration of these opioids is controlled, so neonatal opioid dependency can be lowered, preventing withdrawal symptoms due to abrupt discontinuation. A partial mu-opioid receptor agonist, buprenorphine reduces withdrawal symptoms while diminishing the risk of prolonged opioid exposure. Due to its partial agonist properties, it offers a smooth weaning process as an alternative to traditional opioid treatment [23]. Early physical and occupational therapy initiation can help address motor and developmental delays. Diagnosing the susceptibility of NAS-affected infants to neurodevelopmental challenges and personalized care that supports development is crucial. Engaging the family in the care process is vital. Substance use disorder counseling and treatment for mothers and educating families about NAS create an outline of a supportive environment that optimizes infant outcomes [24].

Long-term effects and developmental outcomes

The impact of NAS is not only limited to the immediate withdrawal phase but also includes the neonate's developmental course through life and longstanding health. The complex relationship between NAS severity, prenatal substance exposure, and subsequent developmental outcomes has gathered researchers' and clinicians' consideration [25]. Neurodevelopmental sequelae involve cognitive and behavioral challenges in later life, such as difficulties in executing functions, hyperactivity, and attention deficits. During NAS withdrawal, disruption in neurotransmitter balance and genetic susceptibilities, if any, results in these cognitive and behavioral issues. NAS-affected children are commonly prone to language and learning delays. Opioid exposure harmfully affects the intricate neural circuit responsible for language and learning developmental achievements. Disturbed brain connectivity, plasticity, and poor caregiving environments lead to these delays [26,27]. Exposure of neonates to a chaotic intrauterine environment impacts socioemotional development. Self-regulatory challenges, attachment difficulties, and emotional dysregulation can be seen in NAS-affected children. Such challenges are aggravated by a continuous cycle of regulatory and emotional struggles and parental substance use. The presence of quality healthcare, access to a sympathetic caregiving environment, and early intervention services can alleviate some of the adverse effects of NAS on socio-emotional development [28,29]. Neurobiological implications include epigenetic modifications due to prenatal opioid exposure that influence gene expression and neurodevelopment. Histone modifications and changes in DNA methylation lead to longstanding effects on neurotransmitter systems and neural circuits [30,31]. All these factors together contribute to the neurobiological sequelae detected in NAS-affected individuals. Vulnerability to substance use disorders is high in NAS-affected individuals in later life. The rehabilitated neurobiology resulting from early opioid exposure may lead these individuals to seek comfort in substances, preserving a cycle of addiction [32].

Multidisciplinary care and support

The general management of NAS demands a multidisciplinary approach due to the wide range of challenges that NAS-affected infants and their families encounter. Collective efforts across psychological, social, and medical fields are crucial to improve outcomes and adopt a supportive environment [33]. Collaboration between neonatologists and clinical pharmacists is pivotal, as pharmacists play a fundamental role in monitoring drug levels and ensuring pharmacological interventions' safety, dose titration, and efficacy. Intrinsic genetic variability and receptor responsiveness in opioid metabolism impact treatment outcomes [34]. Pediatric developmental specialists are crucial in monitoring and identifying neurodevelopmental challenges in NAS-affected infants. Early speech, physical, and occupational therapy interventions improve developmental outcomes. Under family-centered interventions, providing parenting guidance, helping support groups, and tailored counseling groups by psychologists and social workers improve family resilience and adaptive coping tactics [35]. Inclusive treatment is provided to mothers during pregnancy and postpartum, such as counseling and medication-assisted therapy, in collaboration with addiction specialists. Follow-up programs that provide maternal education, neonatal assessments, and counseling on parenting strategies provide an encouraging caregiving environment [36].

Prevention and education

A multilayered approach that includes education and maternal care for preventing NAS is required. Routine screening helps identify pregnant individuals with substance use disorders, enabling early intervention and appropriate support [37]. Wide-ranging prenatal care, including counseling, medication-assisted therapy, and addiction treatment, helps pregnant individuals manage substance use disorders [38]. The combined efforts of addiction specialists, mental health professionals, and obstetricians play an essential role in enhancing maternal and neonatal outcomes. Healthcare providers should be trained in referral pathways and nonjudgmental communication, qualifying them to deliver support without stigma. Public awareness campaigns impact community attitudes and highlight support resources, prevention, and the importance of seeking early care [39]. Providing a stable housing facility and environment with suitable family support services reduces stressors and risks of maternal substance use during pregnancy [40]. Various policy interventions, such as access to addiction treatment, employing prescription drug monitoring programs, and increasing Medicaid coverage, play a crucial role in preventing substance use and NAS [41].

Ethical considerations

Ethical considerations in NAS management incorporate informed consent, treatment decisions, stigma reduction, and the balance between medical intervention and non-pharmacological care. A critical ethical concern arises while balancing a pregnant woman's autonomy with the potential harm to the fetus. Autonomous decision-making, beneficence, and non-maleficence are the ethical principles to be respected. Prenatal care and medication-assisted treatment promote harm reduction strategies, respecting maternal autonomy and fetal interests. Empowering pregnant individuals with complete information to make informed choices prioritizes the neonate's health [30,42]. NAS-affected families often face societal stigma associated with maternal substance use. Healthcare providers have an ethical accountability to create a nonjudgmental, supportive, and caring environment that identifies the challenges faced by these families. Ethical considerations range beyond medical management to include social and emotional support for NAS-affected families. Ethical research concerning pregnant individuals and NAS-affected neonates necessitates careful consideration of risks and benefits [29].

## Conclusions

Under neonatal healthcare, NAS is an intense demonstration of the complex relationship between maternal substance use during pregnancy and the challenges faced by neonates. This comprehensive review has explored various multilayered sections of NAS, highlighting the underlying mechanisms, clinical manifestations, treatment paradigms, ethical considerations, and the domineering role of multidisciplinary care. The core pathophysiology of NAS involves the transplacental transfer of addictive substances, leading to chemical dependence and neuroadaptive responses in the developing fetus. This complex interaction involves receptor sensitization, neurotransmitter dysregulation, and altered neural circuits, ultimately closing in the emergence of withdrawal symptoms postnatally. Clinically, NAS manifests in a diverse array of symptoms ranging from autonomic dysfunction to central nervous system hyperactivity. An accurate assessment is simplified by standardized scoring systems, such as the Finnegan and Neonatal Withdrawal Inventory, assisting in impartial estimation of NAS severity and updating management decisions. Treatment encompasses comprehensive developmental care, non-pharmacological interventions, and pharmacological strategies. Non-pharmacological methods such as swaddling, nutritional optimization, and pharmacotherapy alleviate withdrawal symptoms and promote neonatal stability. NAS-affected infants demand a multidisciplinary approach involving clinical pharmacists, social workers, developmental specialists, addiction specialists, psychologists, and neonatologists. Neurobiological consequences, involving epigenetic modifications, contribute to long-lasting neurodevelopmental sequelae, while vulnerability to substance use disorders remains a concern in later life. Maternal autonomy, stigma reduction, and research ethics are principles of ethics. Balancing maternal decision-making with fetal interests presents a crucial ethical dilemma, highlighting the importance of harm reduction strategies and informed consent. In conclusion, NAS embodies the intricate interplay between maternal substance use and neonatal health. A comprehensive method spanning accurate diagnosis, prevention, longitudinal monitoring, tailored treatment, and ethical considerations is essential to report the multifaceted challenges posed by NAS.

## References

[1] Ross EJ, Graham DL, Money KM, Stanwood GD (2015). Developmental consequences of fetal exposure to drugs: what we know and what we still must learn. Neuropsychopharmacology.

[2] O’Donnell FT, Jackson DL (2017). Opioid use disorder and pregnancy. Mo Med.

[3] Anbalagan S, Mendez MD (2023). Neonatal abstinence syndrome. StatPearls [Internet].

[4] Borrelli KN, Wachman EM, Beierle JA, Taglauer ES, Jain M, Bryant CD, Zhang H (2022). Effect of prenatal opioid exposure on the human placental methylome. Biomedicines.

[5] Jansson LM, Velez M (2012). Neonatal abstinence syndrome. Curr Opin Pediatr.

[6] Abdel-Latif ME, Pinner J, Clews S, Cooke F, Lui K, Oei J (2006). Effects of breast milk on the severity and outcome of neonatal abstinence syndrome among infants of drug-dependent mothers. Pediatrics.

[7] Finnegan LP, Connaughton JF Jr, Kron RE, Emich JP (1975). Neonatal abstinence syndrome: assessment and management. Addict Dis.

[8] Brandt L, Finnegan LP (2017). Neonatal abstinence syndrome: where are we, and where do we go from here?. Curr Opin Psychiatry.

[9] Lester BM, Lin H, DeGarmo DS (2012). Neurobehavioral disinhibition predicts initiation of substance use in children with prenatal cocaine exposure. Drug Alcohol Depend.

[10] Lester BM, Tronick EZ, LaGasse L (2002). The maternal lifestyle study: effects of substance exposure during pregnancy on neurodevelopmental outcome in 1-month-old infants. Pediatrics.

[11] Singer LT, Minnes S, Min MO, Lewis BA, Short EJ (2015). Prenatal cocaine exposure and child outcomes: a conference report based on a prospective study from Cleveland. Hum Psychopharmacol.

[12] Dhaliwal A, Gupta M (2023). Physiology, opioid receptor. StatPearls [Internet].

[13] McCarthy DM, Mueller KA, Cannon EN, Huizenga MN, Darnell SB, Bhide PG, Sadri-Vakili G (2016). Prenatal cocaine exposure alters BDNF-TrkB signaling in the embryonic and adult brain. Dev Neurosci.

[14] Miller-Loncar C, Lester BM, Seifer R (2005). Predictors of motor development in children prenatally exposed to cocaine. Neurotoxicol Teratol.

[15] Hudak ML, Tan RC (2012). Neonatal drug withdrawal. Pediatrics.

[16] Osborn DA, Jeffery HE, Cole MJ (2010). Opiate treatment for opiate withdrawal in newborn infants. Cochrane Database Syst Rev.

[17] Wachman EM, Hayes MJ, Sherva R, Brown MS, Davis JM, Farrer LA, Nielsen DA (2015). Variations in opioid receptor genes in neonatal abstinence syndrome. Drug Alcohol Depend.

[18] Sarkar S, Donn SM (2006). Management of neonatal abstinence syndrome in neonatal intensive care units: a national survey. J Perinatol.

[19] Kosten TR, George TP (2002). The neurobiology of opioid dependence: implications for treatment. Sci Pract Perspect.

[20] Wachman EM, Schiff DM, Silverstein M (2018). Neonatal abstinence syndrome: advances in diagnosis and treatment. JAMA.

[21] Gomez Pomar E, Finnegan LP, Devlin L, Bada H, Concina VA, Ibonia KT, Westgate PM (2017). Simplification of the Finnegan Neonatal Abstinence Scoring System: retrospective study of two institutions in the USA. BMJ Open.

[22] Mangat AK, Schmölzer GM, Kraft WK (2019). Pharmacological and non-pharmacological treatments for the neonatal abstinence syndrome (NAS). Semin Fetal Neonatal Med.

[23] Kraft WK, van den Anker JN (2012). Pharmacologic management of the opioid neonatal abstinence syndrome. Pediatr Clin North Am.

[24] Kocherlakota P (2014). Neonatal abstinence syndrome. Pediatrics.

[25] Yen E, Davis JM (2022). The immediate and long-term effects of prenatal opioid exposure. Front Pediatr.

[26] Bada HS, Das A, Bauer CR (2005). Low birth weight and preterm births: etiologic fraction attributable to prenatal drug exposure. J Perinatol.

[27] Goldfarb SS, Stanwood GD, Flynn HA, Graham DL (2020). Developmental opioid exposures: neurobiological underpinnings, behavioral impacts, and policy implications. Exp Biol Med (Maywood).

[28] Conradt E, Flannery T, Aschner JL (2019). Prenatal opioid exposure: neurodevelopmental consequences and future research priorities. Pediatrics.

[29] Recto P, McGlothen-Bell K, McGrath J, Brownell E, Cleveland LM (2020). The role of stigma in the nursing care of families impacted by neonatal abstinence syndrome. Adv Neonatal Care.

[30] Szutorisz H, Hurd YL (2015). Epigenetic effects of cannabis exposure. Biol Psychiatry.

[31] Kundakovic M, Jaric I (2017). The epigenetic link between prenatal adverse environments and neurodevelopmental disorders. Genes (Basel).

[32] Lester BM, Tronick EZ (2004). History and description of the neonatal intensive care unit network neurobehavioral scale. Pediatrics.

[33] Greene C, Goodman M (2003). Neonatal abstinence syndrome: strategies for care of the drug-exposed infant. Neonatal Netw.

[34] Dilles T, Heczkova J, Tziaferi S (2021). Nurses and pharmaceutical care: interprofessional, evidence-based working to improve patient care and outcomes. Int J Environ Res Public Health.

[35] Bakhireva LN, Holbrook BD, Shrestha S (2019). Association between prenatal opioid exposure, neonatal opioid withdrawal syndrome, and neurodevelopmental and behavioral outcomes at 5-8 months of age. Early Hum Dev.

[36] Suchman N, Pajulo M, DeCoste C, Mayes L (2006). Parenting interventions for drug-dependent mothers and their young children: the case for an attachment-based approach. Fam Relat.

[37] Sterling S, Kline-Simon AH, Wibbelsman C, Wong A, Weisner C (2012). Screening for adolescent alcohol and drug use in pediatric health-care settings: predictors and implications for practice and policy. Addict Sci Clin Pract.

[38] Casavant SG, Meegan T, Fleming M, Hussain N, Gork S, Cong X (2021). Integrated review of the assessment of newborns with neonatal abstinence syndrome. J Obstet Gynecol Neonatal Nurs.

[39] Knaak S, Mantler E, Szeto A (2017). Mental illness-related stigma in healthcare: barriers to access and care and evidence-based solutions. Healthc Manage Forum.

[40] Hirai AH, Ko JY, Owens PL, Stocks C, Patrick SW (2021). Neonatal abstinence syndrome and maternal opioid-related diagnoses in the US, 2010-2017. JAMA.

[41] Deyo RA, Hallvik SE, Hildebran C (2018). Association of prescription drug monitoring program use with opioid prescribing and health outcomes: a comparison of program users and nonusers. J Pain.

[42] Allesee L, Gallagher CM (2011). Pregnancy and protection: the ethics of limiting a pregnant woman’s participation in clinical trials. J Clin Res Bioeth.

